# Lipid class composition of membrane and raft fractions from brains of individuals with Alzheimer's disease

**DOI:** 10.1016/j.bbrep.2019.100704

**Published:** 2019-11-04

**Authors:** Akihiro Kawatsuki, Shin-ya Morita, Naoki Watanabe, Emi Hibino, Yachiyo Mitsuishi, Takuma Sugi, Shigeo Murayama, Masaki Nishimura

**Affiliations:** a Molecular Neuroscience Research Center, Shiga University of Medical Science, Japan; b Department of Pharmacy, Shiga University of Medical Science, Japan; c Department of Neuropathology, Tokyo Metropolitan Institute of Gerontology, Japan

**Keywords:** Alzheimer's disease, Apolipoprotein E, Lipid composition, Brain, Lipid raft, Neurodegeneration

## Abstract

Perturbation of the homeostasis of brain membrane lipids has been implicated in the pathomechanism of Alzheimer's disease (AD). The ε4 allele of the apolipoprotein E gene (*APOE*) confers an increased risk, in a dosage-dependent manner, for brain amyloid-β accumulation and the development of sporadic AD. An effect of the *APOE* genotype on brain lipid homeostasis may underlie the AD risk associated with the ε4 allele. In this research, we examined an effect of *APOE* ε4 on the lipid class composition of crude membranes and raft-enriched fractions of brains. We applied enzymatic reaction-based methods for the quantification of phosphatidylcholine, phosphatidylethanolamine, phosphatidylserine, phosphatidic acid, and sphingomyelin. Our results indicate that brain lipid class composition was neither significantly altered in AD subjects nor affected by the presence of the *APOE* ε4 allele.

## Highlights

• No change was found in the composition of lipid classes of brains with Alzheimer's disease.
• The *APOE* ε4 allele did not affect lipid class composition of the brain membrane or rafts.
• The enzymatic measurement of phospholipids is applicable to brain tissues.

## 1 Introduction

Alzheimer’s disease (AD) is a polygenic neurodegenerative disease characterized clinically by progressive memory loss and, eventually, dementia. The genetic heritability of the sporadic form has been estimated to be 60%–80% [1,2]. The brains of AD patients exhibit loss of synapses and neurons, as well as the presence of neuropathological hallmarks such as senile plaques and neurofibrillary changes. The cores of the senile plaques are composed of aggregated amyloid-β (Aβ) peptide, which is generated from a neuronal transmembrane protein called amyloid precursor protein (APP), and trigger AD pathogenesis.

Altered metabolism of brain membrane lipids has been implicated in the pathogenesis of AD. This hypothesis is based upon multiple lines of evidence. The apolipoprotein E (apoE)-encoding gene (*APOE*) is the strongest genetic risk factor for a sporadic form of AD. In humans, three polymorphic *APOE* alleles (ε2, ε3 and ε4) encode three isoforms carrying amino acid substitutions at residues 112 and 158: apoE2 [Cys^112^, Cys^158^], apoE3 [Cys^112^, Arg^158^], and apoE4 [Arg^112^, Arg^158^], respectively. AD risk is two–four-fold higher for subjects heterozygous for the ε4 allele and eight- to 12-fold for homozygous individuals [3]. Genetic loci close to the *ABCA7*, *TREM2,* and *SORL1* genes, which may be implicated in lipid metabolism, have also been shown to be associated with sporadic AD [4].

The pathogenic potency of Aβ species depends on the length of the C-terminus and the amount of the protein accumulated in the brain. Aβ42 and Aβ43, which have longer C-termini, are more prone to form aggregates, and have more potent pathogenicity. γ-Secretase, an intramembrane aspartic protease, catalyzes the final step in the generation of Aβ, and determines pathogenicity by creating the C-terminus. The local membrane lipid microenvironment has a potent effect on γ-secretase activity and cleavage sites. Increased cholesterol in membrane lipids augments Aβ production and shows a positive correlation with AD development [5]. *In vitro* assays for the γ-secretase cleavage of APP revealed that subtle changes in phospholipid composition greatly modify the activity of γ-secretase. Phosphatidylserine was shown to decrease γ-secretase activity, but increased the relative production of shorter Aβ species, whereas phosphatidylinositol competitively inhibited the γ-secretase cleavage of APP [[6], [7], [8]]. Previous reports have claimed that the specificity of γ-secretase cleavage sites was modified to alter relative production of longer Aβ species in sporadic AD and aged brains [[9], [10], [11], [12]]. In addition to their effect upon Aβ biogenesis, membrane lipids affect Aβ degradation and aggregation. Interactions with cholesterol, gangliosides and phospholipids influence the aggregation of amphiphilic Aβ on the cell membrane [13,14].

The molecular mechanisms underlying the increase in AD susceptibility incurred by the presence of the *APOE* ε4 allele and the modification of γ-secretase activity in the AD brain remain unclear. However, these effects could be closely associated, because apoE plays a pivotal role in the regulation of brain lipid homeostasis. Several previous studies have evaluated brain lipid composition using conventional methods, in which lipids were separated with thin-layer chromatography and quantified by phosphorus analysis of spots. However, these methods have technical difficulties, which have hampered accurate quantification [15]. To examine the lipid class composition of AD brains with or without the ε4 allele, we used enzymatic reaction-based measurements of phospholipids, which were developed by a coauthor of this paper [[16], [17], [18], [19], [20]].

## 2 Materials and methods

### 2.1 Enzymes and reagents

Choline oxidase and lipoprotein lipase were obtained from Wako Pure Chemical Industries (Osaka, Japan). Phospholipase D and glycerophospholipid-specific phospholipase D were purchased from Biomol International (Plymouth meeting, PA). Amine oxidase was provided by Asahi Kasei Pharma (Tokyo, Japan). l-Amino acid oxidase, l-glycerol-3-phosphate oxidase, and sphingomyelinase were obtained from Worthington (Lakewood, NJ), Roche Diagnostics (Mannheim, Germany), and Sigma-Aldrich (St. Louis, MO), respectively. Calf intestine alkaline phosphatase and horseradish peroxidase were obtained from Oriental Yeast (Osaka, Japan). Purified phosphatidylcholine (PC), phosphatidic acid (PA), L-α-palmitoyl-oleoyl phosphatidylethanolamine (PE), phosphatidylserine (PS), and sphingomyelin (SM) were purchased from Avanti Polar lipids (Alabaster, AL). The fluorescent probes Amplex Red (*N*-acetyl-3,7-dihydroxyphenoxazine) and Stop Reagent were purchased from Thermo Fisher Scientific (Waltham, MA).

### 2.2 Human brain tissues

Frozen brain tissues from the temporal cortices of 20 AD patients and 10 age-matched control subjects without neurological disease were obtained from the Brain Bank for Aging Research, Tokyo Metropolitan Institute of Gerontology (Tokyo, Japan). All AD patients fulfilled the criteria of the National Institute of Neurological and Communicative Disorders and Stroke-Alzheimer’s Disease and Related Disorders Associations for probable AD. Formalin-fixed, paraffin-embedded sections were stained with hematoxylin and eosin, Klüver–Barrera's method and Gallyas–Braak's silver impregnation. We also performed immunohistochemistry using antibodies against phosphorylated tau (monoclonal, AT8, Innogenetics, Themes, Belgium), Aβ peptides (monoclonal, 12B2, Immuno-Biological Laboratories, Gunma, Japan), and ubiquitin (polyclonal, Sigma-Aldrich) as previously described [21]. We examined 10 cases heterozygous for the *APOE* ε4 allele and 20 cases negative for the *APOE* ε4 allele. All of the study subjects or their next of kin gave written informed consent for the brain donation, and the Shiga University of Medical Science Review Board approved the study protocol.

### 2.3 Fractionation of membrane lipids and lipid rafts

Frozen brain tissues were homogenized using a motor driven Teflon/glass homogenizer (15 strokes) in four volumes of homogenization buffer (Tris at pH 7.5, 150 mM NaCl, 0.5 mM EDTA). The homogenates were centrifuged at 1500 g to remove nuclei and cellular debris. The supernatants were then ultracentrifuged at 100,000 g for 20 min on a TLA 100.4 rotor (Beckman, Palo Alto, CA, USA). The resulting membrane fraction pellet was resuspended in homogenization buffer.

Fractionation of lipid rafts was performed using buoyant discontinuous sucrose density gradient ultracentrifugation [11] with some modifications. Briefly, 70% by weight of each membrane fraction prepared from 200 mg of brain tissue was suspended in 40% sucrose in MES-buffered saline (25 mM MES at pH 6.5, 150 mM NaCl) containing 1% CHAPSO. Resuspended membrane fractions were placed at the bottom of discontinuous sucrose density gradients of 35% and 5% sucrose and centrifuged at 260,000 g for 4 h. An interface of the 5%/35% sucrose layers was carefully collected and re-centrifuged. The resultant pellet, the lipid raft faction, was washed twice and resuspended in HEPES buffer (25 mM HEPES at pH 7.0, 150 mM NaCl, 5 mM CaCl_2_, 5 mM MgCl_2_).

### 2.4 Measurement of phospholipid class

Lipids in the membrane and lipid raft fractions were extracted using the method of Folch et al. [22], and subsequently dissolved in 1% Triton X-100 solution. The concentrations of PA, PC, PE, PS, and SM were measured as previously described [[16], [17], [18], [19], [20]]. Briefly, PA, PC, PE, and PS were hydrolyzed using phospholipase D to release glycerol-3-phosphate, choline, ethanolamine, and serine, respectively, which were then oxidized with l-glycerol-3-phosphate oxidase, choline oxidase, amine oxidase, and l-amino acid oxidase to generate equimolar H_2_O_2_. SM was hydrolyzed with sphingomyelinase to release phosphorylcholine, which was then dephosphorylated using alkaline phosphatase. Choline was oxidized with choline oxidase to betaine and two H_2_O_2_ molecules. Finally, the production of H_2_O_2_ was assessed by Amplex Red assays using a microplate reader (Infinite M200; Tecan, Männedorf, Switzerland). Standard curves were obtained using purified PA, PC, PS, PE, and SM as described above.

### 2.5 Statistics

Statistical significance was determined using the non-parametric Mann-Whitney *U* test in all experiments.

## 3 Results

### 3.1 Staging of neuropathological hallmarks in the AD brain

Brains collected at autopsy from 20 subjects with clinically-diagnosed AD and 10 non-demented control subjects were used in this study (Table 1). AD subjects included 10 individuals with the *APOE* ε3/ε3 genotype and 10 with the ε3/ε4 genotype, and all control individuals were genotyped as *APOE* ε3/ε3. Using Gallyas–Braak's silver impregnation and tau and Aβ immunostaining, we applied Braak’s staging for neurofibrillary changes (stages I to VI) and senile plaques (stages A to C) for the evaluation of AD-related pathology [23,24]. All 20 of the brains from individuals diagnosed with AD were observed to have a combination of neurofibrillary change of stage IV or above and senile plaque stage C (Table 1), which meet the criteria for definitive diagnosis of AD [21]. Control brains had neurofibrillary changes of stage II or less and senile plaques of stage A or less.

**Table 1:** Demographic and neuropathological findings of subjects.

Age (years)	Sex	Postmortem delay (h at 4 °C)	Neurofibrillary changes	Senile plaques
Non-demented controls (ε3/ε3)				
92	M	15	II	0
89	M	4	II	A
87	M	70	0	0
80	M	3	I	A
82	M	12	I	A
80	M	5	II	A
80	M	8	II	0
88	F	2	I	A
88	M	2	II	A
84	M	3	II	A
AD (ε3/ε3)				
83	F	59	V	C
97	M	3	V	C
86	F	19	V	C
93	M	1	V	C
79	F	3	V	C
93	F	13	V	C
84	M	1	IV	C
80	F	6	V	C
74	M	25	V	C
82	F	8	V	C
AD (ε3/ε4)				
87	M	3	VI	C
86	F	8	V	C
83	M	7	VI	C
87	F	16	V	C
92	F	2	V	C
91	F	47	IV	C
88	M	32	V	C
91	F	11	V	C
79	F	2	IV	C
85	M	7	IV	C

### 3.2 Lipid class composition in membrane fractions from temporal cortex tissues

We used enzymatic methods for measuring the levels of membrane phospholipid classes in the brains. In addition to PC, PE, PS, and SM, we measured PA, which is only a minor component of cell membranes, but is an intermediate for lipid biosynthesis and is involved in the regulation of diverse cellular functions including cell growth, differentiation and migration [25]. To measure lipids, we isolated approximately 200 mg of gray matter from the frozen tissues of the temporal cortex, the region that is most vulnerable to AD pathology. Sufficient membrane lipids were extracted from these tissues to measure the concentrations of PC, PA, PE, PS, and SM. As expected, concentrations of PS, PA, and SM were relatively low compared with those of PC and PE. We estimated and compared the relative concentrations of PA, PE, PS, and SM standardized against PC concentration, because multiple previous reports have indicated that there is no change in the PC content of the brains of individuals with AD [[26], [27], [28]]. Subtle alterations in the relative composition of phospholipid polar head groups have been reported to modify γ-secretase activity [8]. The results indicated no statistically significant difference among AD groups with *APOE* genotype ε3/ε3 and with *APOE* ε3/ε4 and control with *APOE* ε3/ε3 in every lipid class (Table 2). There was a large variance observed in relative SM concentrations in the membrane fraction. This may have been due to microscopic contamination with subcortical white matter or ectopic intracortical myelinated axons, because SM is abundant in the myelin sheath.

**Table 2:** Lipid classes in membrane fractions from brain tissues.

	PA	PC	PE	PS	SM
**Control**	264.0 ± 19.7	607.1 ± 77.5	1079.2 ± 114.5	455.7 ± 60.3	54.2 ± 10.8
**AD ε3/ε3**	258.8 ± 21.8	631.4 ± 70.3	1101.9 ± 103.8	391.2 ± 37.2	64.2 ± 15.1
**AD ε3/ε4**	252.0 ± 19.8	577.0 ± 55.6	997.9 ± 111.0	352.0 ± 35.1	46.2 ± 9.4

	PA/PC	PE/PC	PS/PC	SM/PC
**Control**	0.46 ± 0.02	1.83 ± 0.07	0.78 ± 0.09	0.09 ± 0.01
**AD ε3/ε3**	0.43 ± 0.03 *p* = 0.23	1.80 ± 0.11 *p* = 0.65	0.64 ± 0.03 *p* = 0.36	0.09 ± 0.02 *p* = 0.76
**AD ε3/ε4**	0.45 ± 0.02 *p* = 0.65	1.73 ± 0.07 *p* = 0.60	0.61 ± 0.03 *p* = 0.23	0.07 ± 0.01 *p* = 0.29

Upper table shows concentrations (nmol/g brain tissue, means ± S. E. M.) and lower table shows the ratios to PC (means ± S. E. M., *p* versus the control by Mann-Whitney *U* test).

### 3.3 Lipid class composition in raft fractions from temporal cortex tissues

Lipid rafts, membrane microdomains characterized by high contents of sphingolipids, cholesterol, and saturated fatty acids relative to the surrounding membrane, have been implicated in proteolytic processing for Aβ generation. Altered lipid composition of the rafts may increase the relative amounts of pathogenic Aβ species generated in the brain. A previous study found that γ-secretase activity in the lipid raft fraction was altered in the temporal cortices of AD patients [11]. These findings prompted us to estimate the lipid composition of the isolated lipid rafts from the brains. Using the post-nuclear supernatants of brain homogenates obtained as described above, raft-enriched fractions were prepared by buoyant discontinuous sucrose density gradient ultracentrifugation [11]. There was no statistically significant difference among the three groups (Table 3).

**Table 3:** Lipid classes in lipid raft fractions from brain tissues.

	PA	PC	PE	PS	SM
**Control**	45.7 ± 9.5	127.4 ± 25.3	165.6 ± 34.7	38.1 ± 6.5	22.4 ± 6.5
**AD ε3/ε3**	49.1 ± 10.4	154.3 ± 32.3	212.1 ± 53.3	37.6 ± 6.9	22.9 ± 6.3
**AD ε3/ε4**	38.1 ± 6.5	121.9 ± 22.5	144.4 ± 28.9	34.4 ± 6.5	20.1 ± 3.7

	PA/PC	PE/PC	PS/PC	SM/PC
**Control**	0.37 ± 0.02	1.35 ± 0.10	0.42 ± 0.15	0.16 ± 0.02
**AD ε3/ε3**	0.33 ± 0.02 *p* = 0.29	1.40 ± 0.13 *p* = 0.71	0.31 ± 0.05 *p* = 0.82	0.14 ± 0.02 *p* = 1.0
**AD ε3/ε4**	0.34 ± 0.03 *p* = 0.23	1.17 ± 0.04 *p* = 0.13	0.33 ± 0.06 *p* = 0.88	0.17 ± 0.01 *p* = 0.55

Upper table shows concentrations (nmol/g brain tissue, means ± S. E. M.) and lower table shows the ratios to PC (means ± S. E. M., *p* versus the control by Mann-Whitney *U* test).

## 4 Discussion

In this study, we applied enzymatic reaction-based methods to the quantification of PC, PE, PS, PA, and SM in lipids extracted from human brains. These methods used hydrolyzation and then the oxidization of each phospholipid head group to generate hydrogen peroxide. Each phospholipid has linear stoichiometry with hydrogen peroxide within the biological range. The benefits of these methods include high-sensitivity, low-cost, and simplicity, and can achieve accurate and comprehensive measurements.

We found no significant differences in lipid class composition between the brains of AD patients and non-demented controls. Previous studies have indicated that AD brains have a significant decrease in PE [4,[26], [27], [28], [29]], PC [29], and PI [4,27] compared with control brains. Increases in phospholipid degradation intermediates such as glycerophosphorylethanolamine, glycerophosphorylcholine, and phosphodiesters were detected [[29], [30], [31]], suggesting enhanced catabolism of membrane phospholipids in the AD brains. However, these findings are not universal in the literature; inconsistent results, such as no differences in PC [[26], [27], [28]] and increases in PS [26], have been also reported. All of these studies used conventional methods with thin-layer chromatography and phosphate quantification.

The mechanisms that underlie the link between apoE isoforms and AD are not yet well understood, although both Aβ-dependent and Aβ-independent mechanisms have been suggested. ApoE-containing lipoproteins play a role in lipid delivery, but their role in brain lipid homeostasis remains undefined. A previous study using ^31^P nuclear magnetic resonance revealed that an AD-associated decrease in PC and PE in the brain was more marked in subjects with the ε4/ε3 genotype than in those with ε3/ε3 [32]. The level of phosphoinositol biphosphate (PIP_2_) was also reduced in the brains of ε4 carriers, possibly because of increased expression of a PIP_2_-degrading enzyme named synaptojanin 1 compared with the ε3 counterparts [33]. The brain lipid abnormalities in apoE-null mice shared some similarities with those of AD patients [34]. Pettergrew et al. [35] reported that there were no significant differences in phospholipid composition between ε4-negative (ε3/ε3) and ε4-positive (ε3/ε4 and ε4/ε4) brains with AD. The results of our study supported this result, and suggested that apoE4 does not affect the lipid class composition of brain membranes.

We did not find an effect of the *APOE* ε4 allele on the lipid class composition of raft-enriched fractions from the brains we studied. A previous study using lipid rafts isolated from human frontal cortex in non-demented subjects aged from 24 to 85 years revealed that PE increased with age in women but not in men; SM decreased in men, but not in women; and total polar lipids exhibited significant increases in both sexes [36]. Martín et al. [37], however, reported that the lipid class composition of the lipid rafts from the frontal cortex of brains from individuals with AD was not significantly different from that of healthy subjects, although the lipid rafts from AD-affected brains displayed altered acyl chain saturation. Our result was consistent with their report [37].

The potential limitations of this study include deviations in sexes, and differences in the mean age among subject groups. When selecting brain samples, we prioritized the *APOE* genotype and AD pathology over on sex and age. Additionally, alternative methods of isolation of specialized membrane structures or domains such as synaptosomes may result in different conclusions. We also focused on lipid class on polar head groups in this study, but fatty acyl chain length, saturation, and double-bond isomerization are also important in membrane lipid structure.

## Declaration of competing interest

The authors declare no conflict of interest.
